# Dimensions and Determinants of Trust in Health Care in Resource Poor Settings – A Qualitative Exploration

**DOI:** 10.1371/journal.pone.0069170

**Published:** 2013-07-16

**Authors:** Vijayaprasad Gopichandran, Satish Kumar Chetlapalli

**Affiliations:** School of Public Health, Sri Ramaswamy Memorial University, Tamil Nadu, India; McGill University, Canada

## Abstract

**Background:**

Trust in health care has been intensely researched in resource rich settings. Some studies in resource poor settings suggest that the dimensions and determinants of trust are likely to be different.

**Objectives:**

This study was done as a qualitative exploration of the dimensions and determinants of trust in health care in Tamil Nadu, a state in south India to assess the differences from dimensions and determinants in resource rich settings.

**Methodology:**

The participants included people belonging to marginalized communities with poor access to health care services and living in conditions of resource deprivation. A total of thirty five in depth interviews were conducted. The interviews were summarized and transcribed and data were analyzed following thematic analysis and grounded theory approach.

**Results:**

The key dimensions of trust in health care identified during the interviews were perceived competence, assurance of treatment irrespective of ability to pay or at any time of the day, patients’ willingness to accept drawbacks in health care, loyalty to the physician and respect for the physician. Comfort with the physician and health facility, personal involvement of the doctor with the patient, behavior and approach of doctor, economic factors, and health awareness were identified as factors determining the levels of trust in health care.

**Conclusions:**

The dimensions and determinants of trust in health care in resource poor settings are different from that in resource rich settings. There is a need to develop scales to measure trust in health care in resource poor settings using these specific dimensions and determinants.

## Introduction

Trust is an essential attribute of all human social interactions. [1] A basic level of trust is important for any transaction between human beings. Trust is an important value in health care. [1] The person who is sick seeks the help of the health care provider to heal their illness. This treatment seeking behavior entails a level of trust in the provider. The patient places trust in the provider that they will do the best according to their knowledge and ability to help them heal. Health care involves a certain amount of vulnerability. The doctor has a level of knowledge about the body, its functioning and diseases which puts them in a powerful position. [2] The patient exposes his/her illness to the doctor fully trusting their ability to bring about a cure. The power differential between the doctor and the patient also brings in the potential for exploitation of the patient by the doctor. The patient trusts that the doctor will not resort to any kind of exploitation. Vulnerability, power differential and exploitation are involved in the concept of trust in health care.[3]–[5] Trust in health care is usually defined as a set of expectations that the patient has from the doctor and the health care system to help them heal. This set of expectations includes appropriate diagnosis, correct treatment, non-exploitation, genuine interest in the welfare of the patient and transparent disclosure of all information. [6] Trust has also been defined as an optimistic acceptance of vulnerability in which the patient believes that the doctor will do what is best for the patients. [7] Thus trust is like a forward looking covenant between the doctor and the patient.

Several scholars have tried to describe the dimensions of trust in physician, trust in health facilities, trust in health insurers etc. In an elegant summary of the dimensions of trust in health care Hall et al, describe fidelity, competence, honesty, confidentiality and global trust. Fidelity has been described as respect to the patients’ agency as a person and keeping the welfare of the patient in mind at all stages of the doctor-patient interaction. [7] Competence in diagnosis and treatment has been emphasized as essential for trust building. Being honest and transparent and maintaining confidentiality of the patient information has also been described as dimensions of trust. Global trust is the umbrella term for the aspect of trust which cannot be classified into any of these dimensions. It is intended to capture to holistic aspect of trust which cannot be described by the above mentioned dimensions.

Some empirical data from resource poor settings have revealed certain unique features of trust in health care. A study from Sri Lanka showed that even in the presence of a robust public health system, trust in health care seems to rest on private providers due to perceived quality of care. [8] Another study from Thailand showed that there is a general decline in the trust in public health system due to private practice within the system. [9] In a study from India, it was shown that trust is implicit in the doctor-patient relationship and is largely dominated by the doctor’s opinions and views. [10] In resource poor settings cost of health care, models of health care delivery, accessibility to health care and health care quality are likely to significantly influence trust. Therefore the dimensions and determinants of trust are likely to be different compared to resource rich settings. There is a need to explore the dimensions and determinants of trust in health care in resource poor settings. A good understanding of trust in these settings will help in planning universal health coverage. Goudge and Gilson in their review on researching trust in health care, indicate the importance of qualitative studies to understand the contextual nature of trust before quantitative studies can be done. [11] In keeping with this idea, this qualitative study was done to understand the dimensions and determinants of trust in health care in the urban and rural areas of Tamil Nadu, a state in southern India. Dimensions of trust are those components which formatively or reflectively form the construct of trust, whereas determinants are factors which influence whether a patient has high or low trust. The research aimed at specifically finding the differences in the dimensions of trust between resource poor settings and those that have been previous described from resource rich settings and exploring the various factors that influence the levels of trust in resource poor settings.

### Study Setting

The study was done in Tamil Nadu one of the states in south India. India has a robust public health system which runs through decentralized state budget allocation. With the advent of the National Rural Health Mission, a flagship health system strengthening program of the government of India in 2005, the public health system received a fillip in terms of decentralization, better platforms for community engagement with health care, better accountability mechanisms and greater fund allocation. [12] Alongside this strong public health system there is also a powerful private sector in health care. Private health providers, who deliver health care services for a fee, are the highest contributors to health services in India. There is a growing corporate health industry in the metropolitan cities which provide international quality health services not only to the people in the country but also serve as hubs for health tourism. In addition to the private and public health systems there is a big network of unorganized, unqualified medical practitioners providing all levels of health care. [13] The overall health expenditure is about 4% of the Gross Domestic Product (GDP) and the government budget allocation for health care is less than 1% of the GDP. [14] The remaining health expenditure is largely out of pocket. This leads to significant impoverishment and catastrophic health expenditure is one of the commonest reasons for indebtedness in the country. [15].

The public health system is plagued by system inefficiencies, irregularities, corruption and irrational health practices. The private providers largely remain unregulated and there is high prevalence of irrational care and commoditization of health care.

Tamil Nadu is one of high performing states in India with respect to health indicators. It has one of the well-functioning models of health care in the country but still several pockets especially poor rural areas and migrant urban populations remain largely underserved. [16].

## Methods

The study was done using qualitative research methods. In depth interviews were conducted among members of the community sampled purposively.

### Ethics Statement

The Institution where this study originated has an Institutional Ethics Group which does preliminary review of protocols to decide about the nature of review that they need to go through. The study protocol was reviewed by the Institutional Ethics Group which recommended an expedited review process for the protocol as the ethical risks were minimal. It was approved by the Institutional Review Board and Ethical Committee. Verbal consent was obtained from the participants as most of the respondents were unable to read and write. This was approved by the Institutional Review Board and Ethics Committee. Documentation of the consent was done on a record which was signed by the interviewer and a neutral third party.

### Sampling

To address the main objective of the study the sample were selected from marginalized communities as resource deprivation is most prominent in these communities. Thirty five in-depth interviews were conducted, fifteen among migrant construction workers in and around Chennai, a metropolitan city in Tamil Nadu and sixteen among residents of a rural area in Dharmapuri district of Tamil Nadu and four interviews among primary care doctors catering to the health needs of these participants.

The migrant construction laborers hail from various parts of India, largely from the north Indian states of Orissa, Uttar Pradesh and Jharkhand. They are marginalized because of language barriers, migrant status and poor living conditions. Health access to these migrant construction laborers is very poor. Dharmapuri is one of the districts of Tamil Nadu with poor health indicators. In the surveyed villages the people are agricultural laborers and marginal farmers. In many households the men and women of economically productive age group migrate outside the village to urban areas for work. Since Dharmapuri is a border district in the border between Tamil Nadu and its neighboring state Karnataka, public services are compromised. Though the state of Tamil Nadu has some of the best health indicators in the country, Dharmapuri is among the poor performing districts of Tamil Nadu.

### Interviews

In depth interviews were selected as the methodology for collecting data as the procedure is the most appropriate to gain insights into individual life experiences, trust, and meaning ascribed to trust. [17] The findings are representative of the meanings and experiences of those interviewed. It is highly meaning centric and cannot be generalized to the population. A trained interviewer conducted all the interviews. The construction laborers were approached in their place of residence and the rural participants were spoken to during their visit to primary health centers in their respective villages for health care. The interviews in the rural area were conducted in Tamil language and the interviews with the migrant workers in Hindi. The interviewer started talking to the respondents about health and their perceptions of health. Then the interviewer led the interview towards illness, treatment seeking and choice of health facility. This was followed by discussion about trust in health care. The various aspects of trust, what makes people trust the doctors and what makes people lose trust were explored during the interviews. The interviews last between 30 to 45 minutes each, with some interviews extending up to 2 hours. The interviews were not recorded to prevent the respondents from becoming self-conscious, which often happens among marginalized communities who have an inherent mistrust for research. [18] Notes were taken during the interviews by the interviewer.

### Coding and Analysis

QSR Nvivo software package version 7 was used for coding and analysis of the interviews. The primary researcher read the notes several times and picked three information dense interviews. These interviews were coded by the researcher. The codes were verified and validated by a second researcher after discussions. Following this a coding manual was prepared with detailed descriptions of the codes. The remaining interviews were coded using this manual by the primary researcher. Due to the lack of availability of researchers trained in qualitative data analysis in the team, a third coder could not be engaged. In order to ensure an unbiased third set of coding and to have a fresh re-look at the data set for new perspectives, the primary researcher re-opened the data after a gap of 1 month and redid the coding of the interviews. The differences between the initial coding and recoding were identified and discussed with the second researcher till a consensus was arrived at. The codes were then grouped together into meaningful themes. The identification of themes was largely grounded in the data. But the influences of previous themes present in literature on the identification of these themes cannot be precluded. These pre-existing themes are adequately discussed along with the presentation of the results of the study. The main themes and their interrelationships were assessed based on the interviews. After identifying the themes, the conceptual frameworks were developed and discussed between the primary and second researcher.

### Reflexivity

The primary researcher is a medical doctor by training and so in some of the early interviews could have brought the bias into the interpretation of the results. For example, in some of the early interviews when negative behaviors of doctors and health personnel were pointed out, the primary researcher felt defensive and this could have expressed in his body language and influenced the way the interview went ahead. A reflexivity journal was maintained by the primary researcher in which he reflected on the way certain statements of the respondents were interpreted and the possible alternative interpretations. These were reflected upon and appropriately addressed as memos during the analysis.

## Results and Discussion

The dimensions and determinants of trust in health care are represented in the conceptual table 1.

**Table 1:** Dimensions and Determinants of Trust in Health Care.

Dimensions of Trust	Determinants of Trust
Perceived competence of the doctor/health facility	Comfort with the doctor/health facility
Treatment Assurance – Dependable treatment, dependable at any time of day, dependable irrespective of ability to pay	Personal involvement of the doctor/health care providers
Willingness to accept drawbacks of the doctor/health facility – Tolerant torudeness Tolerant to expensive unaffordable treatments	Doctor/Health Care Provider’s behavior and approach
Loyalty	Economic Factors
Respect	Health Awareness

### Dimensions of Trust in Health Care

The dimensions of trust in health care described here emerged as themes during the analysis of the interviews.

### Perceived Competence of the Doctor/Health Facility

Communities have their unique perceptions about competence of the doctor or the health facility. There are many bases for these competence judgments. In resource poor settings, the lesser health awareness, and lesser access to information leads to highly subjective assessments of competence as compared to resource-rich settings where the higher levels of health literacy and access to information helps people make objective assessments. Community assessment of perceived competence tends to be shared. These competence judgments are often informed by prior personal experiences, opinion of community leaders, and shared opinions of friends and relatives. A common theme that emerged in most rural interviews was that the community perceived that private health facilities are better equipped and qualified to handle serious health conditions compared to public health facilities. An elderly man from a rural area also mentioned that primary health centers in the village are more suitable for women’s problems such as pregnancy, child birth and childhood illnesses. This opinion reflects the high emphasis placed by the public health system on reproductive and child health. While describing the community judgments about trust in the different health facilities an elderly man in a rural area mentioned,

“*if there is some simple problem like knee pain, back pain etc. it is best to go to the Primary Health Center. If there is some other problem we can go to Harur Private Hospital. Harur Private Hospital is a big hospital and all big (sic) doctors are there. So all serious problems can be solved there*”

Doing laboratory tests to make the correct diagnosis is perceived as a competent medical maneuver. In the rural primary health care settings, laboratory tests are not routinely done to arrive at diagnoses because of resource constraints. Therefore doing tests is perceived as a mark of competence. It was obvious in the interviews that the patients did not particularly know what the tests were and why they were being performed. But the acts of being subject to some tests made them trust the physician and the health facility. A young woman who had high fever had come to the primary health center for treatment. During her interview she mentioned,

“*This morning the nurse did blood test for me. The result came within 2 hours and the doctor saw the result and told me that I have typhoid fever. She also advised me to take some more injections and some new tablets. I am feeling much better now and feel confident that I will become better. Without blood tests they wouldn’t have found out my problem correctly*”

Though the knowledge differential between the doctor and patient prevented the patients from making an objective assessment of the quality of their care, they had their respective experiences which helped them form opinions on the competence of the doctor. Some of these opinions are informed by shared judgments of the community, but some are based on their own personal experiences. An elderly man with knee pain who was very happy with the treatment in a primary health center mentioned that the Primary Health Centre provides very good medicines.

“*My entire family gets treatment for any of their problems here (in the primary health center). I refer all my friends and family to come to this PHC for treatment. That is because they give good treatment. I came here for my knee pain and the doctor saw me very well and treated me well. My pain went off within a week. The drugs work very well and we become alright soon.*”

These perceptions of competence of the community are a reflection of the level of trust that they place on the doctor and the health facility. Perception of good level of competence also meant fulfillment of the treatment expectations that the patients had. Thus fulfillment of treatment expectations and competence are closely interlinked dimensions of trust. It was also evident that sometimes despite poor behavioral attributes of the doctors high levels of perceived competence undermined these shortcomings and led to trust.

In previous studies from the resource rich settings, it has been seen that clinical competence is an important component of trust in health care. Goold et al, in their review of various factors affecting the doctor patient relationship in the US have reported that competence is not only an important aspect for trust building; it is also a fiduciary responsibility of the clinician. [19] In the Indian and other resource poor settings, the legal component is not much but this study reveals a significant role of perceived competence in trust building. Competence as a dimension of trust in the western context rests on two important pillars, avoiding medical errors and providing the best possible outcomes of health care. [6], [20], [21] In the current study perceived competence emerged as an important domain but the language in which it was constructed was different. In resource poor settings where public health care facilities struggle to provide even the basic levels of care, expectations are also low. Therefore rather than mentioning medical errors and best possible care in their judgments of competence, the community referred to performing lab tests, making correct diagnosis and giving appropriate medicines in terms of ability to achieve a positive treatment outcome. Unlike the resource rich settings where individuals make judgments about competence of the physician or the health facility, in this study context a large part of competence judgment derived from shared community opinions.

### Assurance of treatment

Assurance emerged as an important dimension of trust. Most respondents related trust to assurance of treatment. They worded trust as the surety or guarantee of some treatment from the doctor. This was narrated as assurance that some basic form of treatment will be provided, assurance of treatment irrespective of ability to pay, and assurance that at any time of the day some kind of treatment will be provided. One of the rural respondents of the interview said,

“*the PHC is like our home. Sometimes we like to eat outside in hotels. But finally we have to come back to our home for home-food. Like that we can go to any private hospital we want and spend any money we want. But if we want something that we can always depend on then we have to come to the Primary Health Center. Whatever happens we can always go there and some kind of treatment will be given. They will not turn us away for want of money*”

This strongly brings about several emotional layers of trust. In resource poor settings eating out is valued as a luxury that is reserved for special occasions. Home food is given high value as a daily source of sustenance, even if it is not as tasty as the food in the hotels or as nutritious. In the cultural context value is attached to home food in terms of guaranteed availability and surety. He equates treatment in the PHC to home food and treatment in private hospitals to hotels and restaurants. In this context, it seems to reflect treatment assurance in a situation of economic deprivation. In some interviews with migrant construction workers the non-availability of the doctor at the timings that they get relieved from their work responsibilities in the evenings came out as an important factor which makes them lose trust in the health system. A woman in a construction site in the urban area said,

“*the doctors are always available in the clinic throughout 24 hours. One of the doctors lives very close to the clinic. So if there is any emergency during any time of the day the doctors can come immediately and help the patient.*”

Another strong narrative that emerged was that the Primary Health Centers provided some basic form of treatment when the patient goes there irrespective of their ability to pay. The private health facilities were reported to have turned away patients because they could not pay. In a migrant interview a man said,

“*last month, one of my colleagues had an injury. We took him to the clinic in Erumavetti Palayam. But the nurses there asked us if we have money. When we said that we did not have money, they just put bandage and sent us away. They refused to put sutures. So we can never trust the doctors and nurses there*”

These uncertainties lead to lesser trust in the health system. Thus financial reasons had a strong underpinning in the dimension of assurance. Financial aspects influence trust in resource rich settings also. In a study in the US, it was shown that fee for service indemnity patients had greater trust in their physician than fee for service managed care patients. [22] But due to the distinct difference in the model of health care in the US and India, the way this aspect of trust is articulated is in terms of treatment assurance irrespective of ability to pay.

### Willingness to Accept Drawbacks in the Doctor

In most of the interviews it was evident that when a trusting doctor-patient relationship was established based on certain domains of trust, the community was willing to overlook or accept the pitfalls in the relationship. The willingness to overlook the pitfalls was reflected in the level of trust that the patients had on the doctor. Though the willingness to tolerate shortcomings may be seen as a consequence of trust, it is also a reflective indicator of the level of trust in which as the willingness to accept shortcomings changes, trust can be perceived to change. In a particular interview with a young woman in a rural area she mentioned about the private doctor practicing in her village and referred to him as a rude person. But she added that whenever she takes her child to this doctor his treatment works very well and so she is willing to accept the rude behavior as she trusts that the doctor intends it in a good way.

“*If the doctor is good it doesn’t matter even if he is rude to us. Even if he scolds us we know very well that he is doing it for our own good. Therefore it is most important for the doctor to be knowledgeable and good.*”

This tradeoff can be seen as a reflection of the level of trust instilled by competence judgments, and a level of treatment assurance. In resource rich situations where there is a choice of doctors, it is reported that about 1/3 of the patients have dissatisfying clinical encounters. Of these a small proportion report complaints. Twenty percent of these are for communication problems and 10% for perceived disrespect or rudeness. [23] But this is very different in resource poor settings. Here behavioral issues such as rudeness are accepted as a tradeoff for competence or other trust dimensions. In resource poor settings, high out of pocket cost of health care is considered as a major deterrent in health seeking. In many of the migrant interviews the fact that came out strongly was that despite high cost, people seek health care with doctors or health facilities in which they place trust. Thus willingness to spend money (which is a scarce resource in the context) on treatment at a particular health facility/doctor emerged as an important dimension of trust. A migrant construction worker said,

“*If anybody who works here becomes sick, we go to the nearest private doctor in Guduvanchery. We like and respect that private doctor. He takes a lot of money. But we prefer that. Even if he takes a lot of money he checks us properly and gives us good and powerful medicines. These medicines help us get better. Even if we spend money, we have to become better. If we don’t become better we cannot work. If we don’t work then what is the use of leaving our homes and migrating here*”

When the vulnerability of the situation increased, this willingness to accept the drawbacks also increased. This was evident from a rural interview with an elderly man with diabetes. This man expressed high level of concern about his illness and seemed to be worried about its consequences as he was the sole breadwinner of this family and their economic condition was poor. When talking about his diabetes doctor in the nearby town he mentioned that despite having to spend a lot of money he feel comfortable because he trusts the doctor to make him better.

“*The medications cost about Rs. 600 per month. I have to literally struggle to make ends meet in my house. Sometimes we even go without any good food to eat. But that is alright. Even if I have to work hard to make that extra money I don’t mind because I know very well that he is a good doctor and it is good for my health if I take the medicines.*”

### Loyalty

Trust was expressed as a sense of loyalty in some of the interviews. When the respondents spoke about some of the doctors or health facilities that they trusted the most, they referred to the fact that whatever the illness may be, they would come to the particular doctor or health facility. They also mentioned that they would not take any other treatment from a higher center without first consulting the particular doctor. This level of loyalty to the doctor is strongly influenced by positive experiences, comfort with the doctor/facility, perceived competence of the doctor/facility. It is also related to the willingness of the patient to accept drawbacks in the treatment given that all other domains of the trust are strong. A previous empirical study showed with the help of structural equation modeling that patient trust is an important predictor of interpersonal relationship and loyalty to the physician. [24] Thus loyalty is an important reflective dimension of trust.

### Respect

Trust was also described as a deep sense of respect for the doctor. In one of the migrant interviews a woman said,

“*the doctor is a very learned man. He has studied for many years to learn this noble art of curing people. I respect the fact that he is more educated and knows a lot of things about disease and treatment*”

The patients perceived the differential in the level of knowledge between them and the doctor and respected the doctor for their knowledge. In addition the other aspects of trust also could increase the level of respect.

### Determinants of Trust in Health Care

The interviews apart from giving an idea about the various dimensions of trust in health care, also gave insights on factors that determined the levels of trust. While perceived competence of the doctor, assurance of treatment, willingness to accept drawbacks in the doctor, loyalty and respect were various dimensions which reflected the trust in the doctor, there were some factors which were identified as determinants of trust.

#### Comfort

Comfort in terms of common language was an important determinant of trust. One of the migrant construction workers said,

“*The only important thing in a doctor is that he should be able to talk to you in your own language and help you out to solve your problem. If you can’t even talk to the doctor properly in your own language, how can you get cured? For people like us who have language barriers, if we have a doctor from our own language we are very happy. If we see a doctor who talks our language we can be sure he will treat us well*”

Apart from comfort of communication, shared language also gave a sense of social connectedness which increased trust. Previous studies have also shown the importance of language, culture and ethnicity in a healthy doctor patient relationship. [25] In the rural participants, the small primary health centers within their own villages which they visited often and were familiar with elicited great amount of trust. The comfort that came from the familiarity seemed to give them the confidence to trust the health facility. An elderly man in one of the rural areas mentioned that familiarity with the facility is very important for him to place trust in a health facility.

“*I like this Primay Health Center. The main reason is because it is very simple. I am very familiar with this PHC because I come here often. I know everybody in this PHC and I know every nook and corner. Because of the familiarity I like to come here.*”

Another aspect of comfort that emerged during the analysis was closely linked to the idea of familiarity. A few respondents mentioned that if someone personally known to them was working in the health facility then they would feel more comfortable with that facility.

#### Personal involvement of the doctor/health care provider

One of the important themes that emerged during the interviews in the rural areas was that the community expected the doctor and health care providers to be involved at a personal level with them. Being recognized by name immediately built the trust in the doctor. An elderly man with diabetes in a rural area, while talking about his physician in the nearby town whom he sees once in 3 months, proudly reported that whenever he goes to the clinic his doctor recognizes him by name and calls him and talks about his personal problems.

A good doctor was referred to by terms which meant, person belonging to us, person from our own family, like our own brother/sister, person who cares about us. The greater the personal involvement of the doctor the greater seemed to be the level of trust. An elderly woman while talking about a young doctor in the primary health center in a rural area mentioned,

“*My husband and I live alone in the village. My sons and daughters all live in Chennai. They come here only for festivals. So we are always looking for somebody to care for us and somebody to talk to us. That young doctor took extra interest and cared for me like my own son. Now I will go and tell my husband and everybody else to come and see this doctor.*”

There is a strong emotional undercurrent in this statement, where the woman tries to replace her missing son with the doctor. The doctor here transcends the social role of provider and fills in another role as the son. This kind of personal interactions between the patients and the doctors seems to be highly valued.

When the health worker takes extra efforts beyond her call of duty she is viewed as a special member of the family and trust builds. One young woman who was talking about the village health worker who helped her father in law when he had tuberculosis mentioned,

“*She regularly visits us at our home and takes care that he takes the medicines regularly. Not only that she also make sure that he gets his monthly sputum tests done. The tuberculosis treatment here is very good. The nurse goes out of her way to help my father in law*”

In the resource-rich context the issue of personal involvement is seen in the lens of boundary crossing and boundary violations. In Australia there has been increased discussion on how the reduction of formalities in medical care has led to increased instances of boundary crossings. Establishment and clear maintenance of professional boundaries is strongly emphasized. [26], [27] This can be contrasted with the research finding in this study where personal involvement with the patient was considered as favorable for trust building. Gift giving and accepting gifts from patients has been a matter of discussion in the context of professional doctor patient relationship. [28] But in settings like India gift giving is an important determinant of trust building. Though it was not empirically explored in this study, it could be seen as an important component of personal involvement of the physician with the patient.

#### Doctor/health care provider’s behavior and approach

In the rural and the migrant interviews certain behavioral factors of the doctor/health provider were highlighted as important for a good health care provider-consumer relationship. Initially behavioral competence was classified as a major dimension of trust during the first iteration of the analysis. But a more important theme that emerged was that perceived technical competence was more important than behavioral competence and people were willing to accept transgressions in behavioral codes as long as their health got better. Therefore we decided that behavior and approach of the doctor played the role of factors determining trust rather than dimensions of trust. Some of the components of the behavior and approach of the health care provider that were identified by the community were:

- Kindness and compassion

“*More than half of the healing takes place because of the kind words of the doctor. Only the remaining is because of the treatment. When we come to a new hospital we are clueless. At that time the doctor should be kind and talk to us patiently. The reason why we prefer private doctors to PHC is because the private doctor talks to us patiently. He spends time with us and checks us up thoroughly. He talks to us and explains everything. That is very important…*” – an elderly man in a rural area.

- Putting themselves in the patient’s shoes and understanding them
- Listening to the patient
- Addressing all doubts and questions

“*The doctor also patiently answers all the doubts and questions that I have. I told him that I am not sure what to eat and what not to eat. He told me that I can eat anything except sweets and meat. He also asked me to reduce the amount of sugar that I put in coffee and tea. He was very kind to me and did not talk rudely at all*” – elderly woman with diabetes in a rural area.

- Explaining the treatment
- Touching the patient

“*Once I had an insect bite in my leg. My leg became very much swollen. It was very painful. So I went to the PHC in Guduvanchery. The doctor there just looked at me and wrote something in the prescription and sent me away. She did not even touch me (holds the hand of the interviewer and gestures). She did not even take my pulse. I felt very upset.*” – a young migrant construction worker.

#### Economic Factors

In low resource settings no discussion of health care trust can be complete without consideration of the cost factor. As noted previously large part of health expenditure in resource poor settings are out of pocket. Sometimes these expenses are catastrophic. In some of the interviews in the migrant construction worker settings it was noted that people had general distrust among doctors who charged huge sums of money. This was typically what Lee et al, would call the ‘trust but verify’ situation. [29] Though they had the doubt whether they were being exploited they also had to trust the doctor because they had no choice. There was coexistence of high trust and high distrust also. A young woman who was a construction laborer was talking about treatment of her daughter’s fever in a nearby private clinic. She said,

“*But nowadays doctors in the private hospitals are asking for a lot of money. When they ask for a lot of money the main problem is that patients cannot believe if the doctor is doing things for the patient’s good or for the doctor’s own benefit*”

The fact that economic dependability emerged as an important dimension of trust was described previously. In most of the interviews economic themes were dominant, and economics was the overall undercurrent cutting across as a factor influencing all the dimensions of trust.

#### Health awareness

The doctors who were interviewed had the opinion that trust in health care is rapidly falling among the community. They attributed this fall in trust to increasing awareness among the people about health and health care. The doctor in the urban area alluded to the ‘google’ culture by which he implied that patients and their care givers were able to readily access health information in the internet and hence asked a lot of questions. Not only this, they also had low levels of trust in the doctor and his/her treatment. In the rural primary health center, the doctor was of the opinion that people have become more aware about health, demand several treatments and ask a lot of questions. They felt that this is counterproductive to healthy treatment relationships. One doctor also mentioned that patients have become empowered and ask questions about quality and availability of care.

“*Nowadays patients are very much empowered. They ask the doctors questions. Last week one day I had to leave early because of some important meeting. The patients asked why the doctor is not there and raised a big issue. Because of that I had to arrange for another doctor to come and run the clinic when I was not there. Nowadays we cannot take the patients for granted. They have become very smart and they ask us questions*”

The interviews with the doctors largely focused on their dissatisfaction with being questioned by the patients. The doctors saw this as a deterioration of trust and perceived that this is an unhealthy trend. Studies from resource rich settings have shown that literacy and awareness have no influence on the level of trust. [30] Therefore the influence of trust on health awareness and health literacy needs to be explored in the resource poor settings to understand this clearly.

This study identified perceived competence, treatment assurance, willingness to accept drawbacks in health care, loyalty, and respect for the doctor as major dimensions which reflect the level trust in health care. Comfort with the doctor, personal involvement of the doctor with the patient, behavior and approach of the doctor, economic factors, and level of health awareness emerged as factors influencing trust in health care.

In a resource rich context, trust in health care is viewed largely based on the rights of the patients. Fidelity, competence, confidentiality, honesty, and global trust are the often discussed dimensions of trust. [7] This has been described as the most important dimension of trust. In our qualitative exploration from resource poor settings it is seen that these same traits are given importance, but they are articulated in words which reflect the need for personal involvement with the doctor.

Confidentiality is highlighted as an important aspect of trust in the resource rich context. [31] This did not emerge as a component in any of the interviews in the current study. Even when specifically probed about the importance of confidentiality of patient information, the community did not mention that it is an important domain of trust. Some researchers have shown that for sensitive issues like abortion, and stigmatizing diseases such as HIV, tuberculosis and leprosy people prefer confidentiality and secrecy in India. A study from south India showed that women preferred confidentiality of information about abortion. [32] But in this study even on deep probing confidentiality failed to emerge as an important theme. Confidentiality would probably feature in the discourse of trust in health care when specific groups such as persons with stigmatizing illnesses are interviewed.

Honesty was another domain of trust which did not emerge as an independent domain. [33], [34] But this came up in the interviews strongly as economic assurance. In the resource poor settings one of the major worries is about money or the lack of it. Therefore economic dependability was seen as honesty. Honesty was perceived in the context of honest economic dealings. Transparency and disclosure of mistakes did not come up during the interviews. This is probably because people had the basic assumption of honesty in diagnosis and treatment but were more worried about economic dishonesty.

A similar exploratory study of trust experiences among patients in primary care setting from California, US revealed technical competence, physician behaviors such as caring, communication, building partnership with the patient, honesty and respect for the patient as domains of trust. [35] But there have been no similar studies in resource poor settings. The current exploratory study strongly points to certain unique differences between the trust in health care in developed and developing country settings. People in resource poor settings, especially the marginalized communities tend to be overwhelmed by sprawling hospital complexes and the maze of rooms and waiting areas in hospitals. Therefore familiarity with small and simple settings gives them a feeling of comfort and this is a very important determinant of trust. Treatment assurance was perceived as being able to walk into a hospital or health facility even without money and having some kind of treatment. Willingness to accept drawbacks in the doctor or health facility was also strongly based on willingness to pay huge sums of money for treatment once trust is established. These were two dimensions where economic factors played an important role in trust building in resource poor settings. The difference between resource rich and resource poor settings is the model of health care delivery and payment mechanisms. Therefore this component of trust is significantly different in the two settings.

The conceptual model for dimensions of trust that is proposed here is largely formative in nature. Each of the components good perceived competence judgments, assurance of treatment, willingness of the patient to accept drawbacks, and respect contributes to trust. In a sense some of these domains could also be viewed as reflective domains of trust especially willingness to accept drawbacks and loyalty.

### Conclusions

This qualitative exploration gives directions for possible domains to be used for development of scales to measure trust in resource poor settings. Development and validation of scales to measure trust in health care in developing country settings would be an important direction to take. This can strengthen the move towards universal good quality health access to all in the developing countries by adequately informing the levels of trust in health care which will influence utilization of the health services.
